# Transcorneal but not transpalpebral electrical stimulation disrupts mucin homeostasis of the ocular surface

**DOI:** 10.1186/s12886-022-02717-z

**Published:** 2022-12-15

**Authors:** Menglu Yang, Anton Lennikov, Karen Chang, Ajay Ashok, Cherin Lee, Kin-Sang Cho, Tor Paaske Utheim, Darlene A. Dartt, Dong Feng Chen

**Affiliations:** 1 Department of Ophthalmology, Harvard Medical School, Schepens Eye Research Institute of Massachusetts Eye and Ear, 20 Staniford St, Boston, MA 02114 USA; 2 Department of Medical Biochemistry, Oslo University Hospital, University of Oslo, Kirkeveien 166, Oslo, 0450 Norway

**Keywords:** Electric stimulation, Dry eye, Corneal epithelial damage, Goblet cells, Corneal epithelial cells, Calcium signaling

## Abstract

**Purpose:**

Transcorneal electrical stimulation (TcES) is increasingly applied as a therapy for preserving and improving vision in retinal neurodegenerative and ischemic disorders. However, a common complaint about TcES is its induction of eye pain and dryness in the clinic, while the mechanisms remain unknown.

**Method:**

TcES or transpalpebral ES (TpES) was conducted in C57BL6j mice for 14 days. The contralateral eyes were used as non-stimulated controls. Levels of intracellular [Ca^2+^] ([Ca^2+^]_i_) were assessed by Fura-2AM. The conductance resistances of the eye under various ES conditions were measured in vivo by an oscilloscope.

**Results:**

Although TcES did not affect tear production, it significantly induced damage to the ocular surface, as revealed by corneal fluorescein staining that was accompanied by significantly decreased mucin (MUC) 4 expression compared to the control. Similar effects of ES were detected in cultured primary corneal epithelium cells, showing decreased MUC4 and ZO-1 levels after the ES in vitro. In addition, TcES decreased secretion of MUC5AC from the conjunctiva in vivo, which was also corroborated in goblet cell cultures, where ES significantly attenuated carbachol-induced [Ca^2+^]_i_ increase. In contrast to TcES, transpalpebral ES (TpES) did not induce corneal fluorescein staining while significantly increasing tear production. Importantly, the conductive resistance from orbital skin to the TpES was significantly smaller than that from the cornea to the retina in TcES.

**Conclusion:**

TcES, but not TpES, induces corneal epithelial damage in mice by disrupting mucin homeostasis. TpES thus may represent a safer and more effective ES approach for treating retinal neurodegeneration clinically.

**Supplementary Information:**

The online version contains supplementary material available at 10.1186/s12886-022-02717-z.

## Introduction

Electrical stimulation (ES) is emerging as a minimally invasive neuromodulatory and neuroprotective therapeutic approach via diverse mechanisms due to its neurotrophic, anti-apoptotic, anti-inflammatory, and vasodilatory activities [1]. It is shown to have beneficial effects on multiple retinal and brain neurological disorders, including retinitis pigmentosa (RP) [2], glaucoma [3], depression [4, 5], seizure [6], Parkinson’s Disease [7], essential tremor [8], and neurological pain [9]. The intensity of the electrical stimulation used in therapeutic ES is usually low (microampere level), which causes minimum to no tissue damage.

In recent clinical studies, ES was found to preserve retinal function in multiple diseases primarily via two routes of application of electrodes: to the corneal surface (transcorneal ES, [TcES]) [2] or the skin of the eyelid (transpalpebral ES [TpES]) [10]. A weekly application of TcES preserved vision in patients with RP [2] and improved retinal function in patients with retinal artery occlusion [11]. TpES treatment also increased visual function in patients with macular degeneration (MD) for up to 4 weeks after the initial ES [10]. While experiencing increased visual acuity from ES, more than half of the study subjects (~ 53%) complained of eye discomfort including dryness and pain after TcES but not after TpES [2, 11]. The discomfort caused by TcES affected the patient’s quality of life during ES treatment and decreased their compliance, discouraging them from receiving proper doses of ES treatment. Currently, little is known about the underlying pathophysiological alterations caused by TcES on the ocular surface and if TcES and TpES have similar effects on inducing eye discomfort or ocular surface damage while delivering electricity to the eye for a therapeutic purpose.

The ocular surface is protected by the tear film, which consists of a thin layer of lipids, electrolytes, water, and glycoproteins, including mucins. This film is spread during blinking to cover the cornea and conjunctiva to form an interface between the mucosal surface and the external environment. Besides the moisturizing and lubricating function, the tear film defends the cornea and conjunctiva against environmental stress, such as bacteria, viruses, pollution, allergens, trauma, and chemicals. Lack of protection of the tear film affects the viability of the corneal epithelial cells [12]. Disturbance of any of the components of the tear-film results in its instability and can cause damage to the underneath epithelium [12, 13].

The tear film contains an aqueous layer generated by the lacrimal gland and accessory lacrimal glands and a mucous layer produced by the epithelial cells of the cornea, conjunctiva, and conjunctival goblet cells [14]. The main component of the mucous layer is mucin, a family of high molecular weight glycoproteins, including the membrane-spanning mucins (mainly MUC1, MUC4, MUC16, and MUC20) [15]. The secreted gel-forming mucin, mainly MUC5AC, is produced by the goblet cells of the conjunctival epithelium. Secreted MUC5AC provides the scaffolding for the mucus layer [15], and together with the transmembrane mucins released by ectodomain shedding and electrolyte and water secretion from the inner layer of the tear film. In particular, failure of mucin production leads to weakening of the barrier function to external pathogens [15]. This study aims to evaluate the potential damage to ocular surface epithelium induced by two ES delivery methods, TcES and TpES, compare their actions on the ocular surface, and assess their conductive resistances to the back of the eye to identify the mode of ES administration that induces optimal therapeutic effects with minimal side effects.

## Materials and methods

### Animals used

Adult female (12-week-old) wild-type C57BL/6 mice were purchased from Jackson Laboratory (Bar Harbor, ME, USA). The mice were kept in a 12-hour light/dark cycle with free access to food and water. Healthy animals without ocular injury were included in the study. All animal experiments were performed following protocols approved by the Institutional Animal Care and Use Committee of the Schepens Eye Research Institute and followed the Association for Research in Vision and Ophthalmology (ARVO) standards of using animals in research. The animal experiments adhered to the ARRIVE guidelines (https://arriveguidelines.org) [16].

### Human conjunctival tissue

Human conjunctival tissues were obtained from Eversight Eye Bank (Ann Arbor, MI, USA) and donated to the eye bank with prior informed consent and authorization of the donor for use in scientific research. The tissues were shipped on ice in preservative media provided by the eye bank. The use of this tissue was reviewed by the Massachusetts Eye and Ear Human Studies Committee and determined to be exempt from ethical approval as it does not meet the definition of research with human subjects.

### Goblet cell culture

Goblet cells from human conjunctiva were grown in organ cultures, as described previously [17]. Conjunctival tissue pieces were placed in culture dishes in RPMI-1640 medium (11-875-085; Thermo Fisher Scientific, Waltham, MA, USA) supplemented with 10% fetal bovine serum (R&D, Minneapolis, MN, USA), 2 mM glutamine (Lonza Group, Basel, Switzerland), and 100 µg/ml penicillin-streptomycin (Gibco, Grand Island, NY, USA). First passage goblet cells were used in all experiments. The cultures were validated by fluorescence microscopy staining with the lectin Helix pomatia agglutinin that detects goblet cell secretory products [17].

### Primary corneal epithelial cell culture

The primary corneal epithelial cells (PKC) were purchased from ATCC: The Global Bioresource Center (PCS-700-010; Manassas, VA, USA). Cells were used at P1 to P2 in all experiments and cultured in serum-free KBMTM-2 Basal Medium (CC-3103, Lonza, Walkersville, MD, USA). The media was supplemented with KGMTM-2 Keratinocyte Growth Medium-2 BulletKit (CC-3107, Lonza, Walkersville, MD, USA) containing Bovine Pituitary Extract 4 µl/ml, recombinant human (rh) Epidermal Growth Factor (EGF) 0.125 ng/ml, Insulin (rh) 5 µg/ml; Hydrocortisone 0.33 µg/ml, Epinephrine 0.39 µg/ml, Transferrin (rh) 10 µg/ml, CaCl_2_ 0.06 mM, and 1% Penicillin and streptomycin.

### Electric stimulation

The electric stimulation in vivo and in vitro was performed with STG4000 (Multichannel Systems, Reutlingen, Germany) pulse generator. The ground electrode was placed on the mouse’s abdomen along with conductiave gel (Spectral 360; Parker Laboratories, Fairfield, NJ, USA) under isoflurane anesthesia. For TcES, the portable electrode probe was applied to the cornea through a conductive gel interface. The portable electrode probe was applied to the contralateral control eye via the conductive gel but without ES. The Spectral 360 conductive gel is reported as a non-ionic compound that relies on hydration for reduced resistance. The gel did not have any significant effect on the corneal surface as both Corneal Fluorescein Staining and phenol-red thread tear production test did not indicate a significant difference (*p* > 0.05) between the naïve eyes and the eyes treated with Spectral 360 conductive gel (daily for 4 min, for 2 weeks; Supplementary Fig. 1). For TpES, the probe was applied to the skin of the orbit. In both TcES and TpES, a biphasic ramp waveform (300 µAmp, 20 Hz) was applied for 4 min daily for 2 weeks, and contralateral eyes were used as controls. Block randomization was performed on selecting the control and experimental eyes. The mice were stimulated in a random order each day.

In primary corneal epithelial cell cultures, the electric current of the biphasic ramp waveform (300 µAmp, 20 Hz) was delivered to the cultures for 30 min daily for 3 days using a c-dish carbon electrode plate (Ion Optix, Westwood, MA, USA). For intracellular [Ca^2+^] detection by fura-2/AM (Invitrogen, Grand Island, NY, USA), the human goblet cells were stimulated once with ES (300 µAmp, 20 Hz) for 60 min.

### Corneal Fluorescein Staining (CSF)

One µL of 2.5% fluorescein (Sigma-Aldrich Corp., St. Louis, MO, USA) was applied to the lateral conjunctival sac, and staining scores were recorded after eye examination using slit-lamp microscopy under cobalt blue light. The punctate staining of the ocular surface was evaluated in a masked fashion and graded as per the National Eye Institute Scoring System (Bethesda, MD, USA), giving a score between 0 and 3 for each of the five areas of the cornea. Each cornea was scored 3 times, and the average score of each time was recorded as the final score. The representative images were acquired using the Micron IV imaging system using an anterior segment imaging rodent slit lamp adaptor with 488 nm excitation and a 520 nm emission filter (Phoenix Micron, Bend, OR, USA).

### Phenol-red thread test

Mice were manually restrained without anesthesia, then a sterile, 75-mm-long phenol-red impregnated thread (Zone-Quick, Osaka, Japan) with 3-mm bent end was placed in the lower fornix of mouse eyes for 15 s. The thread was then removed, and the red portion of the thread was measured from the tip. The length of the color change was recorded.

### Western blot and densitometric quantification

Cells or tissue were washed with cold phosphate-buffered saline (PBS) and sonicated in cold Radioimmunoprecipitation assay buffer (RIPA) buffer containing FAST protease inhibitors (Sigma, St. Louis, MO, USA). The protein concentration of the lysates was determined with the Qubit 4 fluorometer (Thermo Fisher Scientific, Waltham, MA, USA). Total protein (30 µg per lane) was separated by sodium dodecyl sulfate-polyacrylamide gel electrophoresis (SDS-PAGE) using Mini-PROTEAN® 4–20% Precast Gels (Biorad, Hercules, CA, USA) and transferred to 0.45 μm pore-size nitrocellulose membrane. The membranes were blocked with 5% non-fat milk (Biorad, Hercules, CA, USA) at room temperature for 1 h and then incubated overnight at 4 °C with the following primary antibodies (Thermo Fisher Scientific, Waltham, MA, USA): MUC4 (1:500, 35-4900); zonular occludens (ZO-1, 1:1000, 61-7300). β-Tubulin (1:4000; MA5-16308) and β-actin (MA5-15739 1:4000) were used as the loading control. After being washed with PBS-Tween 20 (0.05%; PBST) buffer, the membranes were incubated with horseradish peroxidase (HRP)-conjugated secondary antibody (Biorad, Hercules, CA, USA) Goat Anti-Rabbit IgG (H + L)-HRP (1:2000, 1,706,515) and Goat Anti-Mouse IgG (H + L)-HRP (1:2000, 1,706,516) for 1 h at room temperature. Signals were developed with enhanced chemiluminescence with a Clarity Western ECL Substrate (Biorad, Hercules, CA, USA) and detected with an iBright 1500 gel documentation system (Thermo Fisher Scientific, Waltham, MA, USA). Densitometry analysis was performed using ImageJ software (NIH, Bethesda, MD, USA). The whole uncropped and unprocessed membrane images overlayed with visible light photographs of the membrane with Precision Plus Protein™ Kaleidoscope protein standard ladder (Biorad, Hercules, CA, USA) are presented in Supplementary Figs. 2 and 3.

### Enzyme-linked immunosorbent assay

The whole conjunctiva from both eyes of each animal was removed and incubated in the tear buffer (contains: 1 M NaCl, 0.5 M NaHCO_3_, 1 M KCl, 1 M MgCl_2_, 1 M NaH2PO_4_, 0.5 M HEPES, 1 M CaCl_2_) for 4 h, in the presence of a muscarinic agonist Carbachol (Cch, 10^− 4^ M). The supernatant was then collected, and an ELISA assay (MyBioSource, San Diego, CA) was performed. The kit was used according to the instruction manual, and the resulting absorbances were read at 450 nm using an 800 TS Absorbance Reader (BioTek Instruments, Winooski, VT, USA).

### Mouse cornea whole mounts

Following two weeks of daily TcES or TpES, mice were sacrificed by carbon dioxide inhalation with secondary cervical dislocation. The eyes were immediately enucleated and fixed with 4% paraformaldehyde (VVR Life Science, Radnor, PA, USA) at 4 °C overnight. The corneas were then carefully dissected under the microscope and subjected to blocking and permeabilization in Triton X-100 (0.5%) with 5% normal donkey serum overnight at 4 °C. The samples were then incubated with a primary antibody to MUC4 (1:100; AB1793, Abcam, Cambridge, MA, USA) for 24 h at 4 °C and washed with PBST 3 times for 30 min. After PBST washing, corneal specimens were visualized by donkey anti-mouse IgG (H + L) and Cyanine3 (A10521, 1:1000; Fisher Scientific, Waltham, MA, USA). Specimens were mounted on slides under the microscope, and 4 relaxing cuts were made to flatten the tissue and covered with a ProLong Diamond antifade reagent (Thermo Fisher Scientific, Waltham, MA, USA). The central corneal area was then imaged by confocal microscopy.

### PKC immunofluorescence (IF) staining

PKCs were fixed in 2% paraformaldehyde (VVR Life Science, Radnor, PA, USA) for 5 min, permeabilized by incubation in 0.05% Triton X-100 for 10 min, and blocked with 5% normal donkey serum for 1 h at room temperature (RT). The samples were then incubated with primary antibodies to MUC4 (1:100; AB1793, Abcam, Cambridge, MA, USA) and ZO-1 (1:100; MA5-14568, Thermo Fisher Scientific, Waltham, MA, USA) overnight at 4 °C and washed with PBST. After PBST washing, secondary antibodies Alexa Fluor 488 (A-11,034, 1:1000, Fisher Scientific, Waltham, MA, USA) and Cyanine3 (A10521, 1:1000; Fisher Scientific, Waltham, MA, USA) were applied. The cell nuclei were counterstained by incubation with 4’,6-diamidino-2-phenylindole (DAPI); (1:5000; Sigma, St. Louis, MO, USA). The slides were mounted with a ProLong Diamond antifade reagent (Thermo Fisher Scientific, Waltham, MA, USA) and visualized under fluorescence.

### Imaging

Fluorescent images were obtained with a Leica DMi8 fluorescent microscope and Leica SP8 laser confocal microscope (Leica AG, Wetzlar, Germany). Visible light microscopy images were acquired by Life Technologies EVOS Core XL Imaging System (AMEX1000, Thermo Fisher Scientific, Waltham, MA, USA).

### Measurement of [Ca^2+^]_i _

First passage human conjunctival goblet cells were plated onto 35-mm glass-bottom culture dishes and incubated at 37 °C overnight as described previously [17]. Cells were then incubated for 1 h at 37 °C with Krebs-Ringer bicarbonate buffer containing 119 mM NaCl, 4.8 mM KCl, 1.0 mM CaCl_2_, 1.2 mM MgSO_4_, and 25 mM NaHCO_3_ with 4-(2-hydroxyethyl)-1-piperazineethanesulfonic acid (HEPES) plus 0.5% bovine serum albumin containing 0.5 µM fura-2/AM (Invitrogen, Grand Island, NY, USA), 8 µM pluronic acid F127 (Sigma-Aldrich, St. Louis, MO, USA) and 250 µM sulfinpyrazone (Sigma-Aldrich, St. Louis, MO, USA) for 1 h. Before Ca^2+^ measurements, cells were washed with KRB-HEPES containing sulfinpyrazone. Ca^2+^ measurements were conducted using a ratio imaging system (In Cyt Im2; Intracellular Imaging, Cincinnati, OH, USA) using excitation wavelengths of 340 and 380 nm and an emission wavelength of 505 nm.

### Rose bengal dye uptake by primary corneal epithelial cultures

The rose bengal staining and detection were performed as a modification of the protocol reported in the literature [18]. PKC cells at P2 were seeded in 6 well plates and grown to confluency in KBMTM-2 media with a supplement pack, followed by electrical stimulation (Ramp, 20 Hz, 300 µA) for 30 min per day for 3 days. The cultures were washed with PBS and incubated in Rose Bengal (Sigma-Aldrich, St. Louis, MO, USA) at 1 mg/ml for 90 s, followed by three washes with PBS. Representative images were then taken, and the uptake of rose bengal dye by PKC was quantitatively ascertained. Three hundred microliters of 100% methanol were added to cell monolayers and then centrifuged at 5,000* g* for 5 min to remove cellular debris. The methanol extracts were transferred to 96-well plates, and optical density was detected at the rose bengal absorbance peak of 570 nm using an 800 TS Absorbance Reader (BioTek Instruments, Winooski, VT, USA).

### Resistance recording

An oscilloscope (Hantek Electronic Co, Shandong, China) was used for resistance recording. The machine was calibrated using a 1 M$\varOmega$ Resistor (E-Projects, Michigan, USA) before each measurement. Mice were anesthetized with ketamine (100 mg/kg) and xylazine (20 mg/kg). Under a surgical microscope, the negative probe was connected to a 30G needle penetrating the retina, while the positive probe was connected to the cornea or orbital skin, where the ES probe was placed. Conductive gel (Spectral 360; Parker Laboratories, Fairfield, NJ, USA) was applied to the positive probe to be consistent with the ES procedure. The resistance between the cornea and the retina or between the orbital skin and the retina was recorded 3 times for each eye, and the average value was used for analysis.

### Statistical analysis

The data are presented as the fold-increase above basal or average ± standard deviation (SD). The student’s *t-*test was used in 2 group comparisons, and One-way ANOVA with Dunnett correction was used in multiple group comparisons. The *p*-value of less than 0.05 was considered statistically significant.

## Results

### TcES induces corneal epithelial damage after 14 days of continuous application in mice

**Fig. 1:**
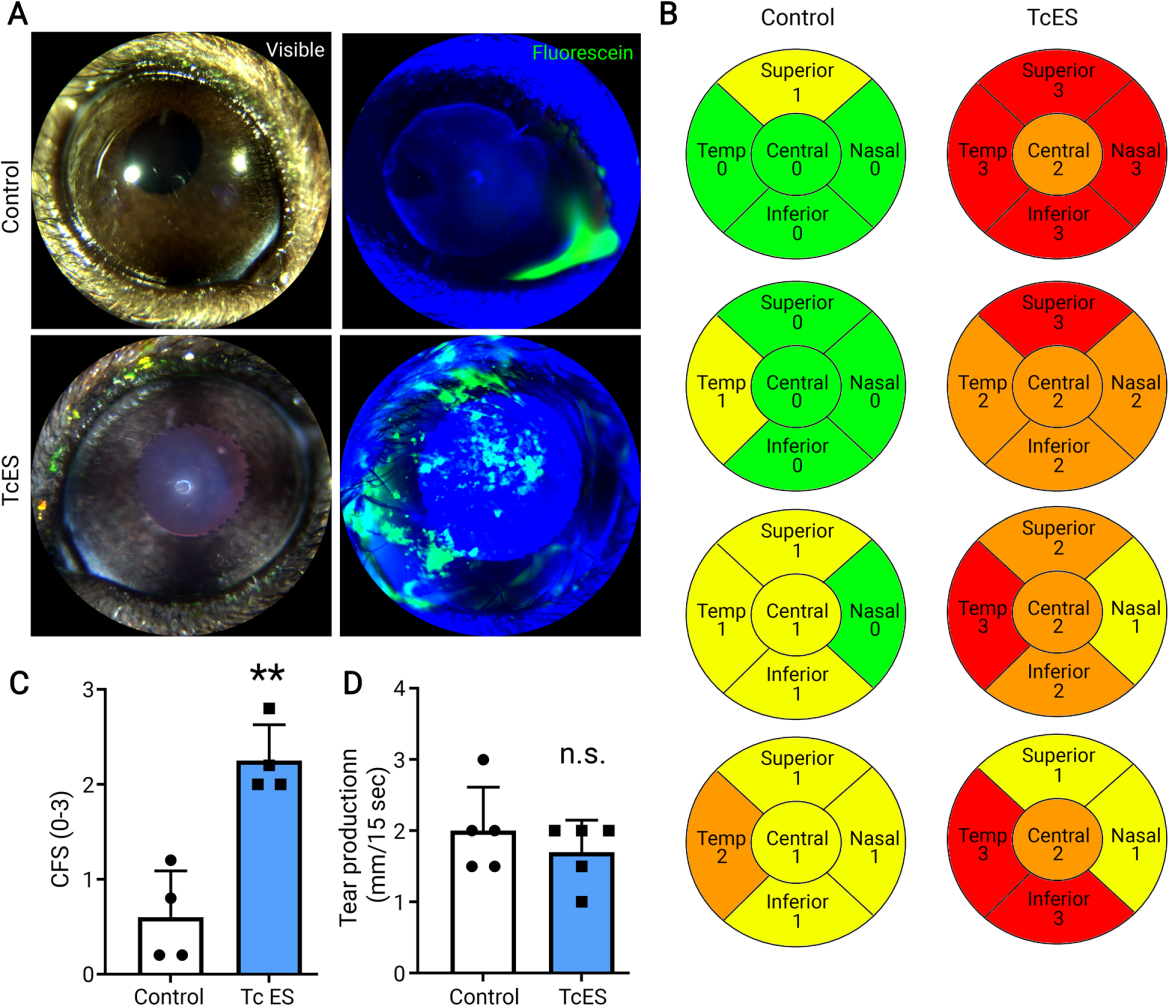
TcES induced corneal epithelial damage after 14 days of continuous application in mice. Sodium fluorescein dye was applied to each eye 14 days after TcES, and images were taken under slit lamp microscopy before (**A** left panel) and after (**A** right panel) sodium fluorescein dye application. Representative images were shown in A. In eyes without TcES, minimum to no fluorescein stain was presented (**A** top panel); in the eyes treated with TcES, medium to severe fluorescein stain was presented (**A** bottom panel). The fluorescein stain was scored (**B**), and the mean value of the scores was analyzed (**C**). The tear production from each eye was measured and plotted in (**D**). Each data point is the mean value of one cornea. ** *p* < 0.01; n.s. *p* > 0.05 (*n* = 4 eyes/group)

The right eye of each animal underwent a short TcES session of 4 min per day for 14 consecutive days through a conductive gel. The same conductive gel and the electric probe were applied to the left eye without ES as vehicle control. On day 14, a corneal fluorescein staining test was performed to assess ocular surface damage. The slit lamp observation showed severe corneal fluorescein staining in the TcES eye, while minimum to no staining in the contralateral eyes (Fig. 1A and B). Quantification data revealed a significantly increased fluorescein staining score in the TcES eyes compared to the control eyes (*p* = 0.0018, Fig. 1C), implicating the induction of dry eye.

We next determined whether the corneal epithelial damage was due to the lack of tear production (aqueous deficiency). Phenol-red thread was inserted into the lower conjunctival sac of the mouse eyes after 14 consecutive days of TcES or sham treatment, and the length of the color change section was measured to indicate the tear production rate of the eye. No significant difference was found in the tear production rate between the TcES eye and the control (*p* = 0.40, Fig. 1D). This result suggests that the dry eye caused by TcES is not due to aqueous deficiency but may be caused by the disturbance of other tear film components.

### TcES decreases transmembrane mucin production of the corneal epithelium in vivo

**Fig. 2:**
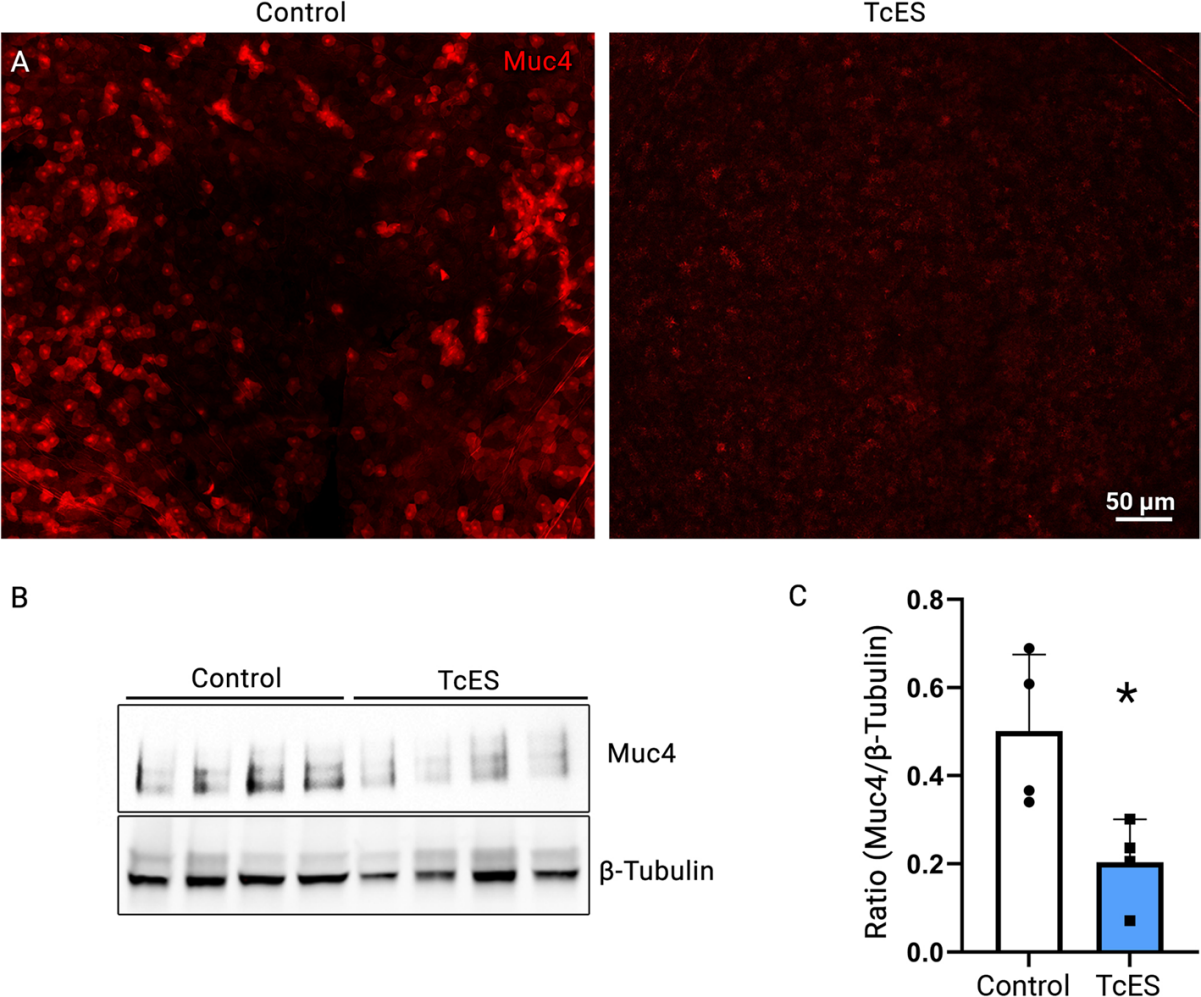
Decreased immunoreactivity of MUC4 was detected in the corneas subjected to TcES. The mice corneas were harvested after 14 days of ES and IF on the cornea. The whole mount was conducted in non-treated eyes (**A** left panel) and TcES-treated eyes (**A** right panel). The immunoreactivity of MUC4 was detectable throughout the cytoplasm in corneal epithelial cells located in the top layer of the stratified squamous epithelium (**A**). WB analysis was conducted using corneas undergone TcES (*n* = 4) or those of the contralateral control eyes (*n* = 4) (**B**). A single band at 150 kDa was detected in all samples (**B**). The ECL signal from each band was normalized to ß-tubulin, and the quantification result was plotted in (**C**). * *p* < 0.05. Original blots/gels are presented in Supplementary Fig. 2

To investigate the mechanism of the TcES-induced corneal epithelial damage, we examined the transmembrane mucin of the corneal surface. TcES and control eyes were collected from the animals after 14 days of ES, and corneal whole-mount staining was performed using primary antibodies against MUC4, which is commonly expressed by both human and mouse corneal epithelial cells. The immunoreactivity of MUC4 was present on the apical layers of the corneal epithelium in control eyes but was markedly reduced in TcES eyes (Fig. 2A). This was confirmed by Western Blot analysis using corneal tissues treated with TcES and the untreated contralateral eyes (Fig. 2B). Quantification results showed a near 60% decrease in MUC4 protein signal in the TcES group compared to the contralateral eye (*p* = 0.025, Fig. 2C), indicative of TcES-induced corneal MUC4 deficiency.

### Electrical stimulation directly disturbs transmembrane mucin production and the tight junctions of the corneal epithelium in vitro

**Fig. 3:**
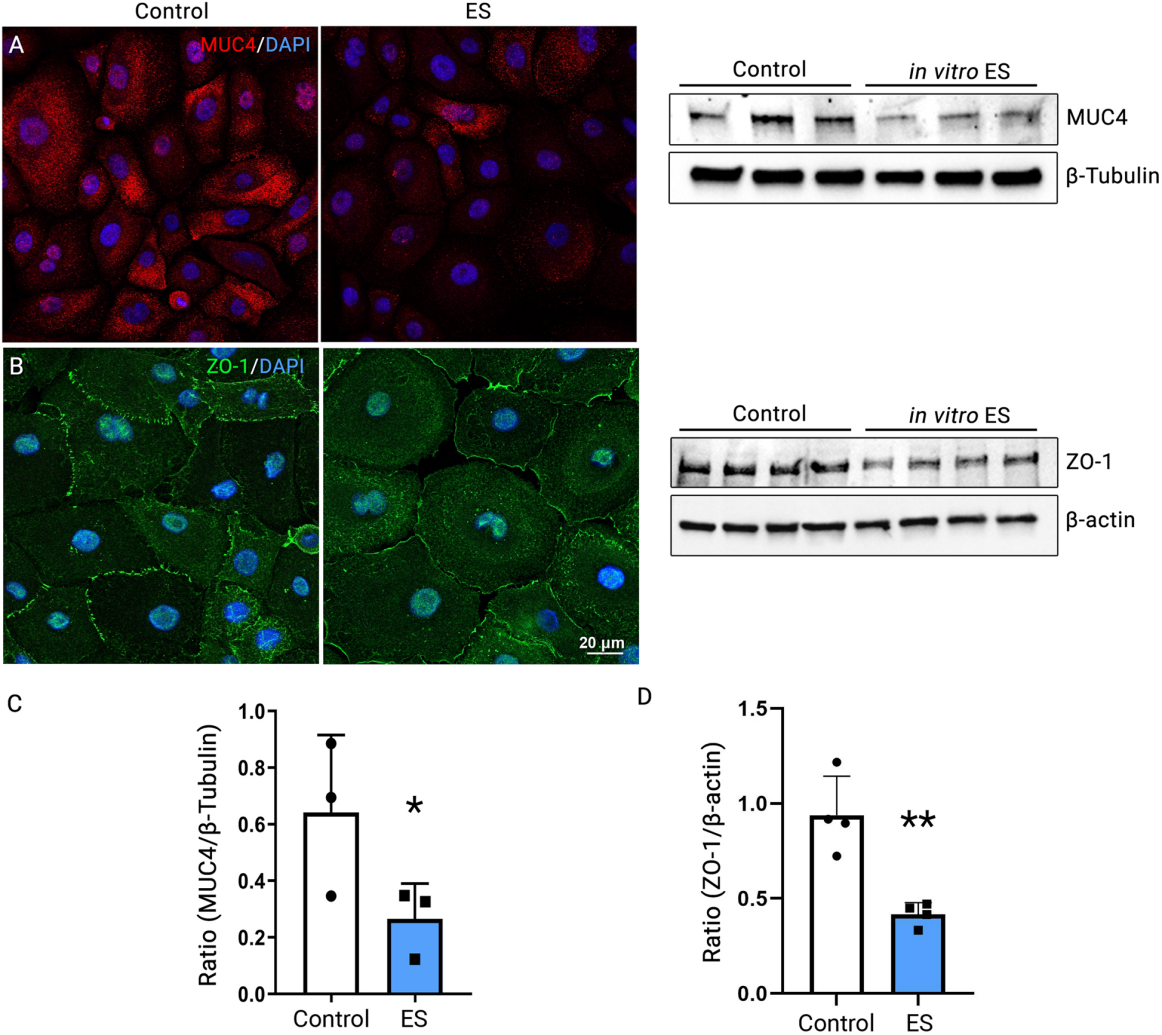
ES inhibited MUC4 and ZO-1 expression in primary corneal epithelial cells. Primary corneal epithelial cells were cultured on coverslips till confluency. ES was applied 30 min each day for 3 days, and immunofluorescence microscopy was performed with cultured goblet cells using antibodies against MUC4 (**A**) and ZO-1 (**B**). The panels on the left are representative images from control, while the panels on the right are representative images from cells treated with ES. Protein samples were collected from each group’s cell pellets, and Western Blot analysis was performed using antibodies against MUC4 (**A**) and ZO-1 (**B**). For MUC4, 3 lanes on the left indicate proteins extracted from control, and 3 lanes on the right indicate proteins extracted from the ES-treated cells. For ZO-1, 4 lines on the left indicate proteins extracted from control, and 4 lines on the right indicate proteins extracted from the ES-treated cells. The quantified relative abundance of MUC4 (**C**) and ZO-1 (**D**) is plotted and analyzed. * *p* < 0.05. ** *p* < 0.01. Original blots/gels are presented in Supplementary Figs. 2 and 3

To study the direct actions of ES on the corneal epithelium, we employed ES in PKC cultures. PKC were cultured until the monolayers formed. ES was applied to the monolayer PKC cultures for 30 min each day for 3 days. As PKC monolayer cannot be sustained as a confluent culture for more than a day, longer ES time was employed to compensate for the shortened treatment cycle than used in mice in vivo. Cells were then fixed and analyzed by immunolabeling using primary antibodies against MUC4. MUC4 immunoreactive signal (red) presented as a punctate pattern throughout the cytoplasm in non-ES groups; a reduced MUC4 signal was observed in cells treated with ES (Fig. 3A). As the disruption of tight junctions between corneal epithelium cells is another important hallmark of dry eye [19], we also investigated the tight junction of PKC using ZO-1 as a marker. ZO-1 immunoreactive signal (green) presented as a serrulate pattern outlining each corneal epithelial cell in the non-ES group; reduced fluorescence signal and blurry lining were observed in cells after ES treatment compared to controls, supporting that ES disrupted tight junctions of PKC (Fig. 3B).

Western blotting analysis and quantification of MUC4 expression showed a near 60% decrease in MUC4 protein levels in the ES group compared to control (Fig. 3A, right panel; Fig. 3C, *p* = 0.047). Similarly, the level of ZO-1 protein decreased by 55.55 ± 11.40% in ES groups compared to the control (Fig. 3B right panel; Fig. 3D, *p* = 0.0028).

We further examined the general transmembrane mucin production or function in cultured PKC using Rose bengal. Rose bengal is an organic anionic dye that stains damaged corneal epithelial cells. The transmembrane mucin expressed on the epithelial cell surface prevents the uptake of rose bengal dye by cells; thus, more rose bengal staining correlates with reduced mucin expression and function [18]. We observed a higher level of rose bengal staining in cells that underwent ES than in controls. We extracted rose bengal dye from the cells and measured the fluorescence intensity at an excitation wavelength of 570 nm using an ELISA reader (BioTek Instruments, Winooski, VT, USA). The fluorescence intensity was significantly higher in extraction from ES-treated cells than in controls (*p* = 0.015, Supplementary Fig. 4). These results supported that ES applied to the corneal epithelial cells negatively impacts the production or function of transmembrane mucin.

### TcES negatively regulates the secretion of secretory mucin by inhibiting intracellular Ca^2+^ ([Ca^2+^]_i_) signaling in goblet cells

**Fig. 4:**
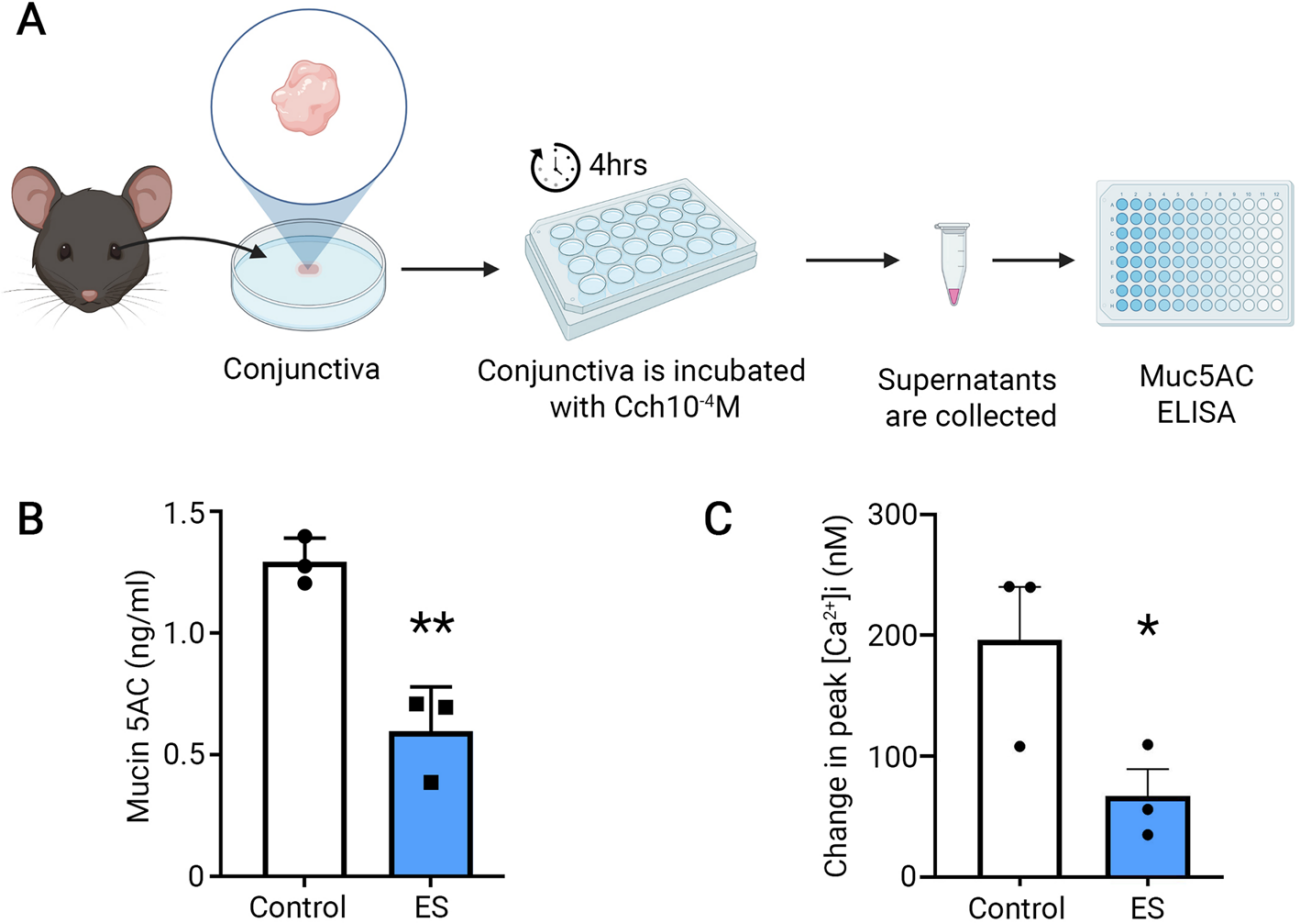
TcES negatively regulates the secretory mucin release by inhibiting intracellular [Ca^2+^]_i_ signaling in conjunctival goblet cells. After 14 days of TcES, the whole conjunctiva (*n* = 3 for each group) of the mice are dissected and incubated in the tear buffer for 4 h in the presence of Cch (10^− 4^ M). After 4 h incubation, the supernatant of each conjunctiva was collected, and the level of MUC5AC was detected using ELISA (**A**). The amount of MUC5AC was plotted in (**B**). Primary human conjunctival goblet cells were treated with ES for 1 h, and then the [Ca^2+^]_i_ mobilization was induced by Cch (10^− 4^ M) and measured in cells treated with or without ES. Each data point indicates a single individual assay. * *p* < 0.05. ** *p* < 0.01

In addition to the decrease in transmembrane mucin caused by ES, we also determined the effect of TcES on the secretory mucin, mainly MUC5AC, from the goblet cells of the conjunctiva in vivo. After 14 days of TcES in vivo, the whole conjunctiva of each animal was dissected and incubated in tear buffer (contains: 1 M NaCl, 0.5 M NaHCO_3_, 1 M KCl, 1 M MgCl_2_, 1 M NaH2PO_4_, 0.5 M HEPES, 1 M CaCl_2_) for 4 h in the presence of a muscarinic agonist carbachol (Cch, 10^− 4^ M). Cch mimics mucin secretion induced by the efferent parasympathetic nerve that could occur normally. After a 4 h incubation, the supernatant of the conjunctiva was collected, and the amount of MUC5AC was detected using ELISA (Fig. 4A). We noted that conjunctival tissue removed from the TcES eyes showed a significant decrease in the amount of secreted MUC5AC than tissue from the contralateral control eyes (*p* = 0.0043, Fig. 4B).

Because the increase in [Ca^2+^]_i_ is usually involved in stimulating the secretion of MUC5AC from the conjunctival goblet cells [20], we investigated goblet cell [Ca^2+^]_i_ responses after ES. Human primary goblet cells taken from individual donors were seeded in glass-bottomed dishes. Fura2 AM was loaded into the cultured cells 2 h before the [Ca^2+^]_i_ was measured. One hour after Fura-2 AM addition, ES or control probes without ES were applied to the cultured cells for 1 h. At the end of the treatment, cells were washed and stimulated with Cch (10^− 4^ M) to mimic the physiological stimulation by the efferent parasympathetic nerves. The [Ca^2+^]_i_ level over time was recorded, and the change in peak [Ca^2+^]_i_ was calculated by subtracting the average of the basal value from the peak [Ca^2+^]_i_ value. In non-ES treated cells, the addition of Cch (10^− 4^ M) increased the [Ca^2+^]_i_ to 196.2 ± 43.98 nM above the baseline; while in ES treated cells, the addition of Cch (10^− 4^ M) increased the [Ca^2+^]_i_ level to only 67.05 ± 22.24 nM, which is significantly nearly 3x lower than the non-ES treated control (*p* = 0.029, Fig. 4C). These results indicate that TcES negatively regulated the production and release of secretory mucin by decreasing the [Ca^2+^]_i_ response of conjunctival goblet cells.

### TpES does not induce corneal epithelial damage nor alter the amount of corneal MUC4

**Fig. 5:**
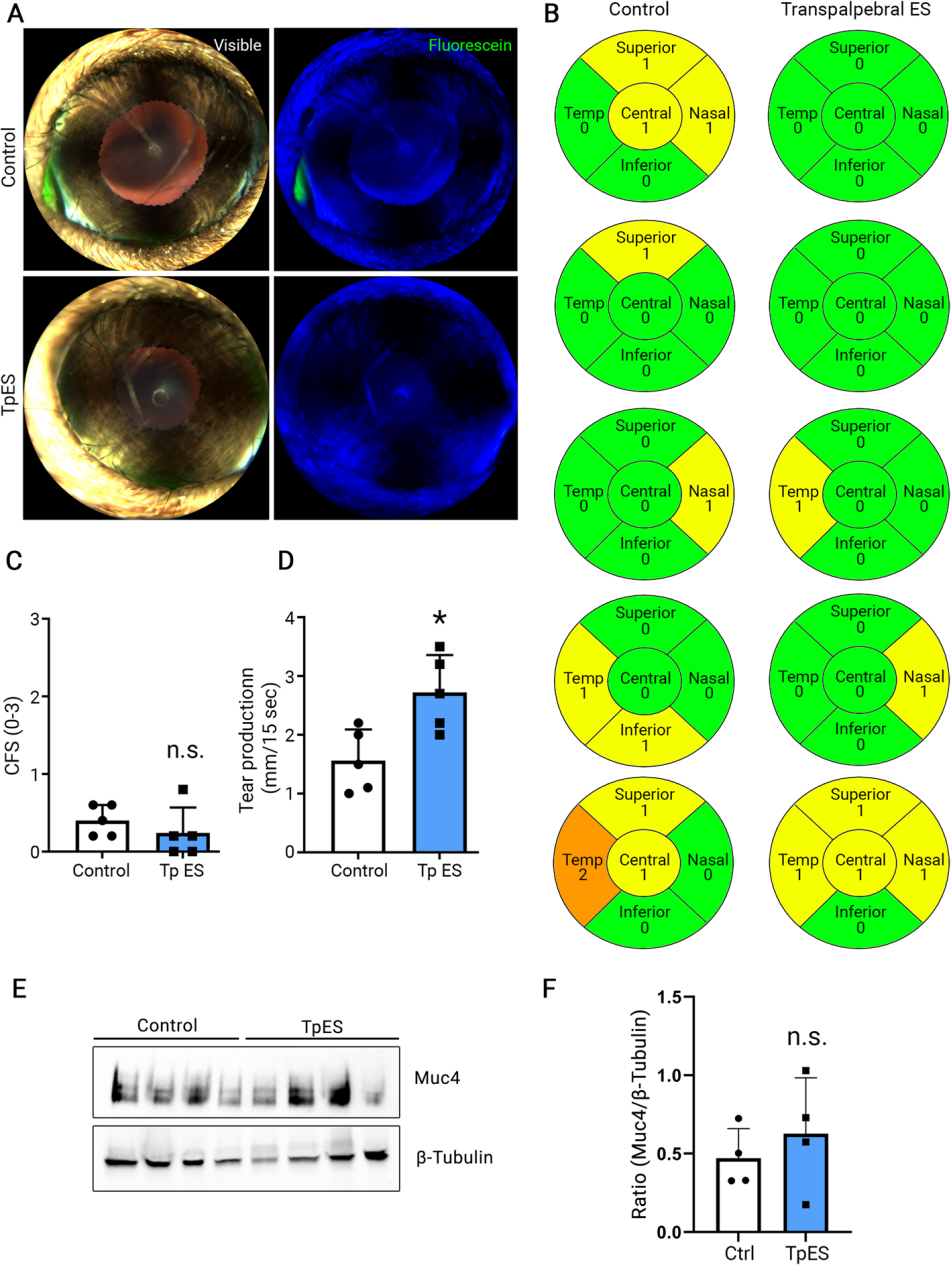
TpES did not induce corneal epithelial damage while increasing tear production after 14 days of continuous application in mice. Sodium fluorescein dye was applied to each eye after TpES 4 min per day for 14 days, and images were taken under slit lamp microscopy before (**A** left panel) and after (**A** right panel) sodium fluorescein dye application. Representative images were shown in A. Minimum to no fluorescein stain was present in both control (top) and TpES eyes (bottom). The fluorescein stain was scored (**B**), and the mean value of the scores was analyzed (**C**). The tear production from each eye is plotted in (**D**). Each data point is the mean value of one cornea. * *p* < 0.05; n.s. *p* > 0.05. WB analysis was conducted using corneas undergone TpES (*n* = 4) or those of the contralateral control eyes (*n* = 4) (**E**). The ECL signal from each band was normalized to ß-tubulin, and the quantification result was plotted in (**F**). n.s. *p* > 0.05. Original blots/gels are presented in Supplementary Fig. 3

We examined the effect of TpES on the ocular surface. In a similar design, TpES of the same parameter or control probes without ES was applied to the mouse eyelid for 4 min per day for 14 consecutive days. On day 14, corneal fluorescein staining testing was performed. The slit lamp observation showed minimum to no corneal fluorescein staining in both the TpES and control eyes (Fig. 5A). The staining score showed no difference between TpES and control eyes (*p* = 0.38, Fig. 5B and C).

Moreover, phenol-red threat measurement of tear production showed a significant increase in tear production in the TpES eyes compared to the controls (*p* = 0.014, Fig. 5D). In addition, Western Blot analysis of MUC4 amount was conducted on corneas removed from animals treated with TpES in one eye and no stimulation of the contralateral eye (Fig. 5E). No significant difference was observed in the amount of MUC4 in the TpES corneas compared to corneas from the control eyes (*p* = 0.47, Fig. 5F). In summary, TpES does not cause ocular surface damage nor alter MUC4 expression.

### TpES bypasses the cornea and delivers a higher electrical current to the retina than TcES

To understand why TpES does not cause ocular surface damage and compare the efficacy of electrical current delivery to the retina by TpES and TcES, we assessed the conductive resistances from the stimulation site to the retina.

**Fig. 6:**
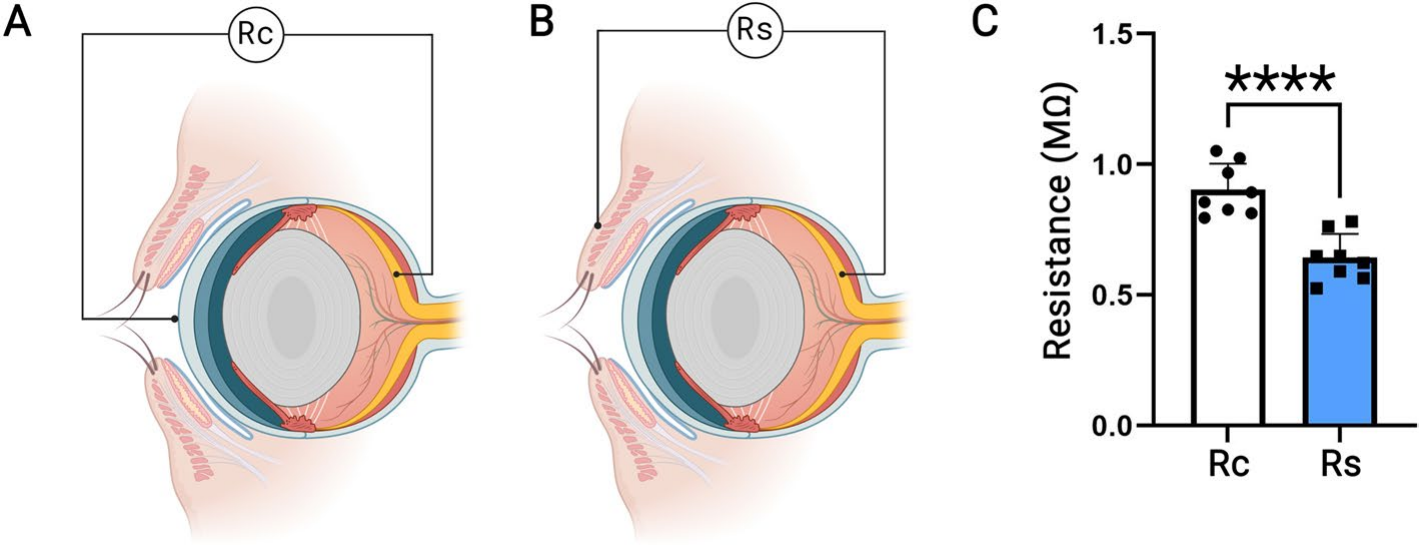
TcES presented higher conductance resistance to the retina than TpES. The resistance from the surface of the cornea to the retina (Rc; **A**) and the total resistance from the orbital skin to the retina (Rs; **B**) of the same eye were measured, and each measurement was plotted in (**C**). **** *p* < 0.0001

We employed direct current (DC) from the corneal surface to the retina (Rc; Fig. 6A) or the orbital skin of the upper eyelid to the retina (Rs; Fig. 6B) of the same eye and measured the total conductive resistance. The result shows that Rc is 0.90 ± 0.035 MΩ, which is significantly higher than Rs of 0.64 ± 0.032 MΩ (*p* < 0.0001, Fig. 6C), suggesting that TpES is more effective than TcES for delivering electricity to the retina. To understand how much of the electricity delivered via TpES is likely to pass through the cornea, we measured the resistance between the TpES site (orbital skin) and the cornea. We noted that the resistance between the cornea surface and the TpES site is 2.54 ± 0.33 MΩ, ~4x higher than the resistance measured from the skin to the retina. Given that the conductive resistance from the orbital skin to the cornea is > 4 folds larger than that to the retina, we can conclude that no more than 20% electric current goes through the cornea under TpES. Together, our data suggest that while TcES induces ocular surface epithelial damage, TpES may present a better alternative that effectively delivers electricity to the back of the eye without interfering with the mucin homeostasis of the ocular surface.

## Discussion

We demonstrated that TcES induces ocular surface epithelial damage in mice by inhibiting both transmembrane and secretory mucin production, while the electrical current evoked by TpES bypasses the cornea and does not cause such damage. As mucin in the tear film not only plays a role in hydrating and lubricating the eye surface but also trapping and removing pathogens, TcES-induced ocular surface epithelial damage may increase cornea’s susceptibility to microorganisms such as *Pseudomonas. aeruginosa (P.a.) *[21]. Considering the increasing popularity of minimal-invasive ES in the treatment of retina and brain neurological disorders, our data suggest that TpES is superior to TcES because of its smaller conductive resistance (higher efficacy of electricity delivery) and the absence of corneal side effects.

More than 20 genes of mucins have been identified in humans that can be categorized into transmembrane and secretory [22]. In the current study, we chose MUC4 to represent mucins of this category because it is expressed by both humans and mice. We observed a decreased MUC4 signal in cornea, decreased protein in cultured primary corneal epithelial cells after ES, and decreased ZO-1. The effect of ES on protein synthesis has been reported in various cell types [23–25]. Recently we have demonstrated that not only neurons, but non-excitable cells such as retinal Müller glia cells [26] and microglia cells can response to electric stimulation [27]. Studies have implicated that the phosphoinositide 3-kinase (PI3K)-Akt (protein kinase B/PKB)-mammalian target of rapamycin (mTOR) pathway may play a role in ES-induced responses [28]. mTOR is a ubiquitously-conserved serine/threonine kinase critical in conducting growth signals and metabolism [29]. mTOR can form two protein complexes: mTORC1 and mTORC2 [29]. mTORC1 senses the oxygen, growth factor, energy, and stress levels, while mTORC2 mainly regulates the cytoskeleton [30]. This pathway can be regulated by various molecules, including phosphatase and tensin homolog (PTEN). PTEN negatively regulates the phosphatidylinositol 3,4,5-triphosphate (PIP3), which inhibits the Akt/mTOR signaling [31]. Previous studies have reported a decreased expression of PTEN induced by electric stimulation in primary human keratinocytes [25], which is consistent with our findings in the BV-2 microglial cell line [27]. In that same study, we observed decreased expression in numerous genes of different types after ES, such as cytoskeletal, ion transport, energy metabolism, and cell junction. Therefore, we speculate that the decreased expression of MUC4 and ZO-1 is due to the inhibitory effect of ES on protein synthesis.

In the current study, we found that TcES reduce the expression of the secretory gel-forming mucin MUC5AC by conjunctival goblet cells. MUC5AC is the main secretory mucin of the ocular surface [32, 33].

Given that gel-forming mucins lubricate the ocular surface while trapping the debris or pathogens from the outside environment from binding to the epithelial cells [34, 35], the reduced MUC5AC secretion caused by TcES may increase the vulnerability of the ocular surface to pathogens in addition to contributing to dry eye. While complications of the ES therapy are not sufficiently studied, TcES may likely increase the chances of infectious bacterial or viral conjunctivitis.

Mucin secretion by conjunctival goblet cells is tightly related to [Ca^2+^]_i_, and we observed that ES abolished the [Ca^2+^]_i_ surge induced by Cch. These findings indicate that the inhibition of MUC5AC secretion by ES is likely accomplished by [Ca^2+^]_i_ signal inhibition. Interestingly, the unpublished data of our group has observed a universal inhibition of [Ca^2+^]_i_ signaling after ES in multiple cell types. Since [Ca^2+^]_i_ is an important signaling molecule that facilitates numerous cell functions, including cell-cell interaction, migration, activation of immune cells, and energy metabolism, TcES may affect the ocular surface immune system in many other ways. We can infer from our results that TcES causes discomfort and damages the ocular surface epithelial barrier, which, although not yet reported clinically, increases the susceptibility to infection.

Although it is counterintuitive that TpES is more efficient than TcES for delivering electrical current to the back of the eye, our data provided a plausible explanation: the conductive resistance of the Rs is smaller than the Rc. The eyeball is surrounded by the sclera, which comprises of dense collagen fibers, leading to high resistance. However, the nerves and blood vessels entering the retina through the optic nerve head are connected to the other pole through the vasculature system. The resistance in the vascular system is relatively low (approximately 300Ω) [36], making it easier for the electric field to propagate through the vasculature. As the vasculature also supplies the skin, TpES may offer less resistance to the electrical current traveling to the retina than TcES. Special attention should be paid to the skin’s condition because factors such as the hydration level, hair distribution, and micro-incision could dramatically affect the skin’s resistance [36]. The skin should be well prepped and hydrated to get the best effect of TpES.

In contrast to causing ocular surface damage, we observed an increased tear production in TpES eyes compared to no ES in the control eyes. This increased tear production by TpES may be due to facial nerve stimulation since the facial ganglion is located underneath the TpES site in mice [37]. The lacrimal gland is innervated by the trigeminal nerve and the facial nerve, and the parasympathetic fibers from the facial nerve of the lacrimal gland stimulate tear production [38]. Therefore, TpES or ES to the facial nerve may be a potential therapeutic approach for dry eye.

There are limitations in our current measurement of the electrical current. Although the direct measurement of the electric current in eye tissues is beyond the scope of our equipment, we plan to sketch the electric field around the orbit and corroborate the results with recent efforts by Lee et al., who are using computer simulations to estimate ocular tissue conductivity [39, 40].

Considering that heat generated during the stimulation may potentially contribute to dry eye development, we assessed temperature changes in the eye or the conductive gel before and after the ES, using an infrared thermometer with 0.1˚C resolution. However, no temperature change was observed in the eye or conductive gel during the ES.

In this study, a frequency of 20 Hz was used in all experimental settings, an optimal ES frequency based on our previous report [41]. Theoretically, the increased frequency will increase electrical conductance, leading to more electrical current delivered to the body while increasing the risk of tissue damage. The current frequency of 20 Hz is safe for biological applications. In the future, the threshold of ES frequency may need further study.

## Conclusion

TcES, but not TpES, causes ocular surface epithelial damage and decreased corneal barrier function. TpES may present an efficient and safe ES delivery method to the retina. Given that the TcES electrode is more difficult to manufacture than the eye patches used for TpES, TpES also offers a convenient and less invasive approach to ES therapeutic application.

## Supplementary Information

**Additional file 1: Supplementary Figure 1.** (A) Corneal Fluorescein Staining scores and (B) tear production of eyes non-treat (Naive) or treated with Spectral 360 conductive gel (Gel). Each data point is the mean score of one cornea. n.s. *p* > 0.05 (*n* = 5 eyes/group). **Supplementary Figure 2.** Whole western blotting membrane images of (A) mouse Muc4 (~130 kDa) and β-tubulin (~55 kDa) loading control (B). The cropped region used in Figure 2 is indicated by the black squares; (C) human MUC4 (~270 kDa), several fragments with a similar sample expression pattern as the target band were detected at ~35 kDa and ~20 kDa, suggesting protein fragmentation during blotting process; (D) β-tubulin (~55 kDa) loading control. The cropped region used in Figure 3 is indicated by the black squares. Images are acquired in automatic exposure determination before signal oversaturation. **Supplementary Figure 3.** Whole western blotting membrane images of (A) human ZO-1 (~260 kDa) and β-actin (~42 kDa) loading control (B). The cropped region used in Figure 3 is indicated by the black squares; (C) mouse Muc4 (~130 kDa) and (D) β-tubulin (~55 kDa) loading control. The cropped region used in Figure 5 is indicated by the black squares. Images are acquired in automatic exposure determination before signal oversaturation. **Supplementary Figure 4.** Rose Bengal staining on primary corneal epithelial cells. Primary corneal epithelial cells were cultured on coverslips till confluency. ES was applied 30 min each day for 3 days and Rose Bengal 1 mg/ml was applied for 3 min, and images were taken under brightfield microscopy. (A) is a representative image of control and (B) is a representative image of cells treated with ES. After imaging, the cells were washed and Rose Bengal dye was extracted by methanol. The optical density values of methanol extracts were determined at 570 nm (C). *n*=5 (independent cultures) * *p* < 0.05.

## Data Availability

All data generated or analyzed during this study are included in this published article and its supplementary information files.

## Abbreviations

[Ca^2+^]_i_: Intracellular Ca^2+^
Akt: Protein kinase B/PKB
CSF: Corneal fluorescein staining
MD: Macular degeneration
mTOR: Mammalian target of rapamycin
MUC: Mucin
PI3K: Phosphoinositide 3-kinase
PIP3: Phosphatidylinositol 3,4,5-triphosphate
PKC: Primary human corneal epithelial
PTEN: Phosphatase and tensin homolog
RP: Retinitis pigmentosa
TcES: Transcorneal electrical stimulation
TpES: Transpalpebral electrical stimulation
ZO-1: Zonular occludens - 1
