# Supplementary material for: Transcorneal but not transpalpebral electrical stimulation disrupts mucin homeostasis of the ocular surface
Source: BMC Ophthalmol. 2022 Dec 15;22:490. doi: 10.1186/s12886-022-02717-z (PMC9756492; doi:10.1186/s12886-022-02717-z)
Supplement: Supplementary file 1 — Additional file 1: Supplementary Figure 1. (A) Corneal Fluorescein Staining scores and (B) tear production of eyes non-treat (Naive) or treated with Spectral 360 conductive gel (Gel). Each data point is the mean score of one cornea. n.s. p > 0.05 (n = 5 eyes/group). Supplementary Figure 2. Whole western blotting membrane images of (A) mouse Muc4 (~130 kDa) and β-tubulin (~55 kDa) loading control (B). The cropped region used in Figure 2 is indicated by the black squares; (C) human MUC4 (~270 kDa), several fragments with a similar sample expression pattern as the target band were detected at ~35 kDa and ~20 kDa, suggesting protein fragmentation during blotting process; (D) β-tubulin (~55 kDa) loading control. The cropped region used in Figure 3 is indicated by the black squares. Images are acquired in automatic exposure determination before signal oversaturation. Supplementary Figure 3. Whole western blotting membrane images of (A) human ZO-1 (~260 kDa) and β-actin (~42 kDa) loading control (B). The cropped region used in Figure 3 is indicated by the black squares; (C) mouse Muc4 (~130 kDa) and (D) β-tubulin (~55 kDa) loading control. The cropped region used in Figure 5 is indicated by the black squares. Images are acquired in automatic exposure determination before signal oversaturation. Supplementary Figure 4. Rose Bengal staining on primary corneal epithelial cells. Primary corneal epithelial cells were cultured on coverslips till confluency. ES was applied 30 min each day for 3 days and Rose Bengal 1 mg/ml was applied for 3 min, and images were taken under brightfield microscopy. (A) is a representative image of control and (B) is a representative image of cells treated with ES. After imaging, the cells were washed and Rose Bengal dye was extracted by methanol. The optical density values of methanol extracts were determined at 570 nm (C). n=5 (independent cultures) * p < 0.05. [file 12886_2022_2717_MOESM1_ESM.pdf]

Transcorneal but not transpalpebral electrical stimulation disrupts mucin homeostasis of the ocular surface

(Supplementary data)

Menglu Yang, MD, Ph.D[1#], Anton Lennikov, MD, Ph.D[1,3#], Karen Chang, Ph.D[1,3], Ajay Ashok, Ph.D[1,3], Cherin Lee[1], Kin-Sang Cho, Ph,D[1], Tor Paaske Utheim, MD, Ph.D[1,2,3], Darlene A. Dartt, Ph.D[1*], Dong Feng Chen, Ph.D[1*]

[1]Schepens Eye Research Institute of Massachusetts Eye and Ear, Department of Ophthalmology, Harvard Medical School, 20 Staniford st, Boston, Massachusetts, United States, 02114;
[2]Department of Ophthalmology, Oslo University Hospital, University of Oslo, Kirkeveien 166, 0450, Oslo, Norway; utheim2@gmail.com
[3]Department of Medical Biochemistry, Oslo University Hospital, University of Oslo, Kirkeveien 166, 0450, Oslo, Norway; utheim2@gmail.com
[#] These authors contributed equally to this study.

*** Correspondence:**
Dong Feng Chen, Schepens Eye Research Institute, 20 Staniford St, Boston, MA 02114, +1(617)-912-0100; dongfeng_chen@meei.harvard.edu;
Darlene A. Dartt, Schepens Eye Research Institute, 20 Staniford St, Boston, MA 02114, +1(617)-912-0100; Darlene_Dartt@meei.harvard.edu

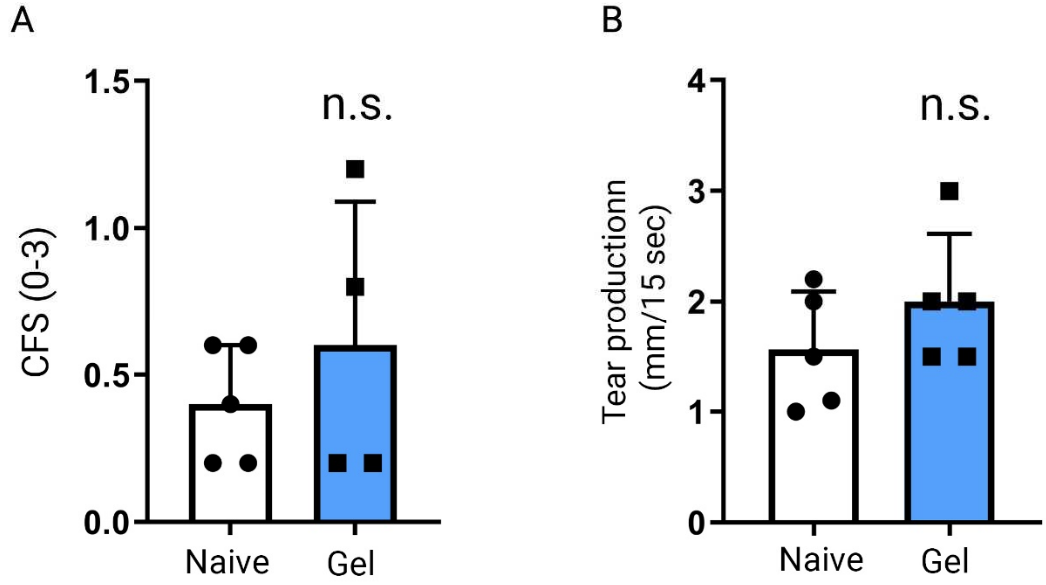

Supplementary Figure 1: (A) Corneal Fluorescein Staining scores and (B) tear production of eyes non-treat (Naive) or treated with Spectral 360 conductive gel (Gel). Each data point is the mean score of one cornea. n.s. $p > 0.05$ (n = 5 eyes/group).

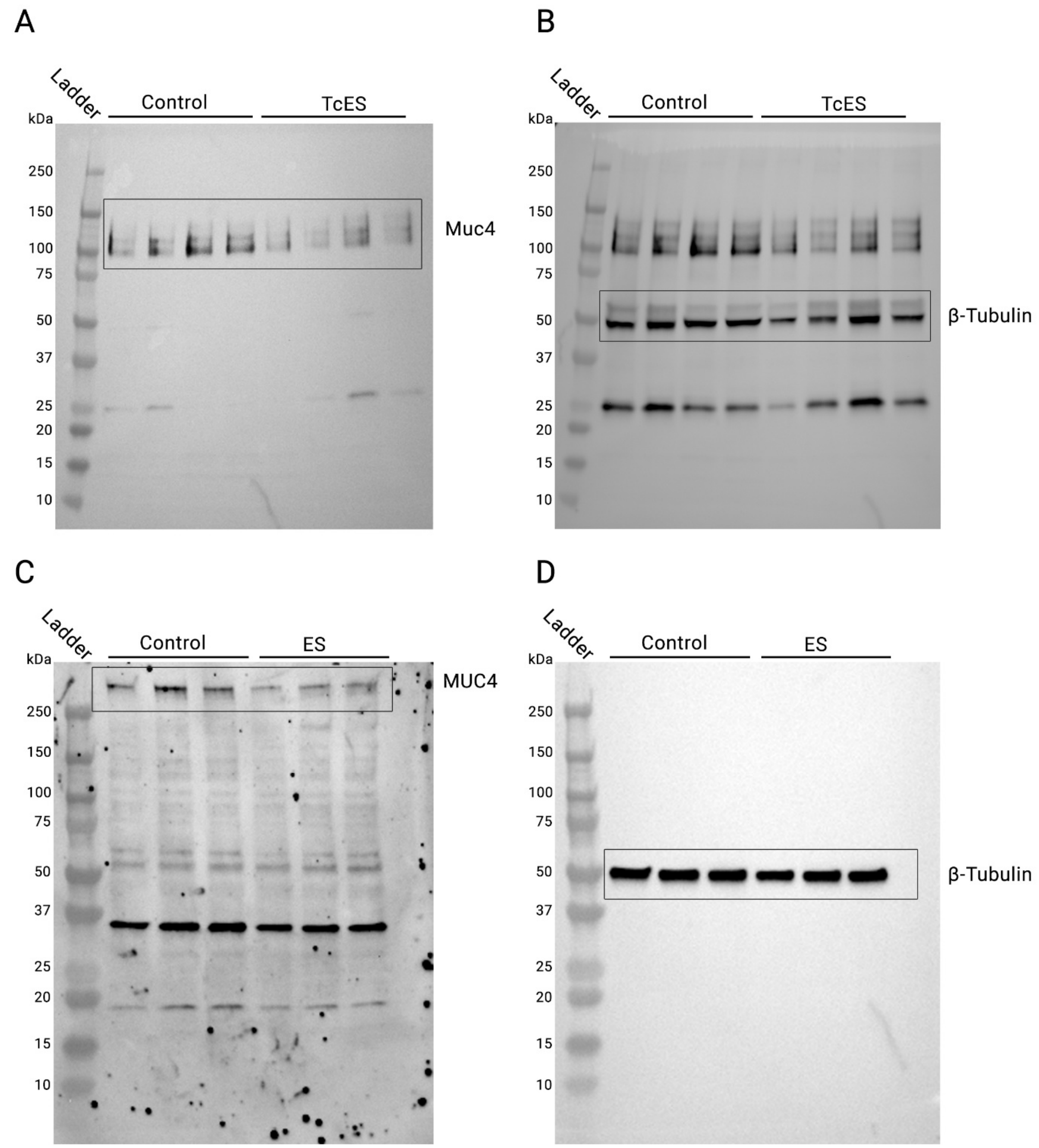

Supplementary Figure 2: Whole western blotting membrane images of (A) mouse Muc4 (~130 kDa) and β-tubulin (~55 kDa) loading control (B). The cropped region used in Figure 2 is indicated by the black squares; (C) human MUC4 (~270 kDa), several fragments with a similar sample expression pattern as the target band were detected at ~35 kDa and ~20 kDa, suggesting protein fragmentation during blotting process; (D) β-tubulin (~55 kDa) loading control. The cropped region

used in Figure 3 is indicated by the black squares. Images are acquired in automatic exposure determination before signal oversaturation.

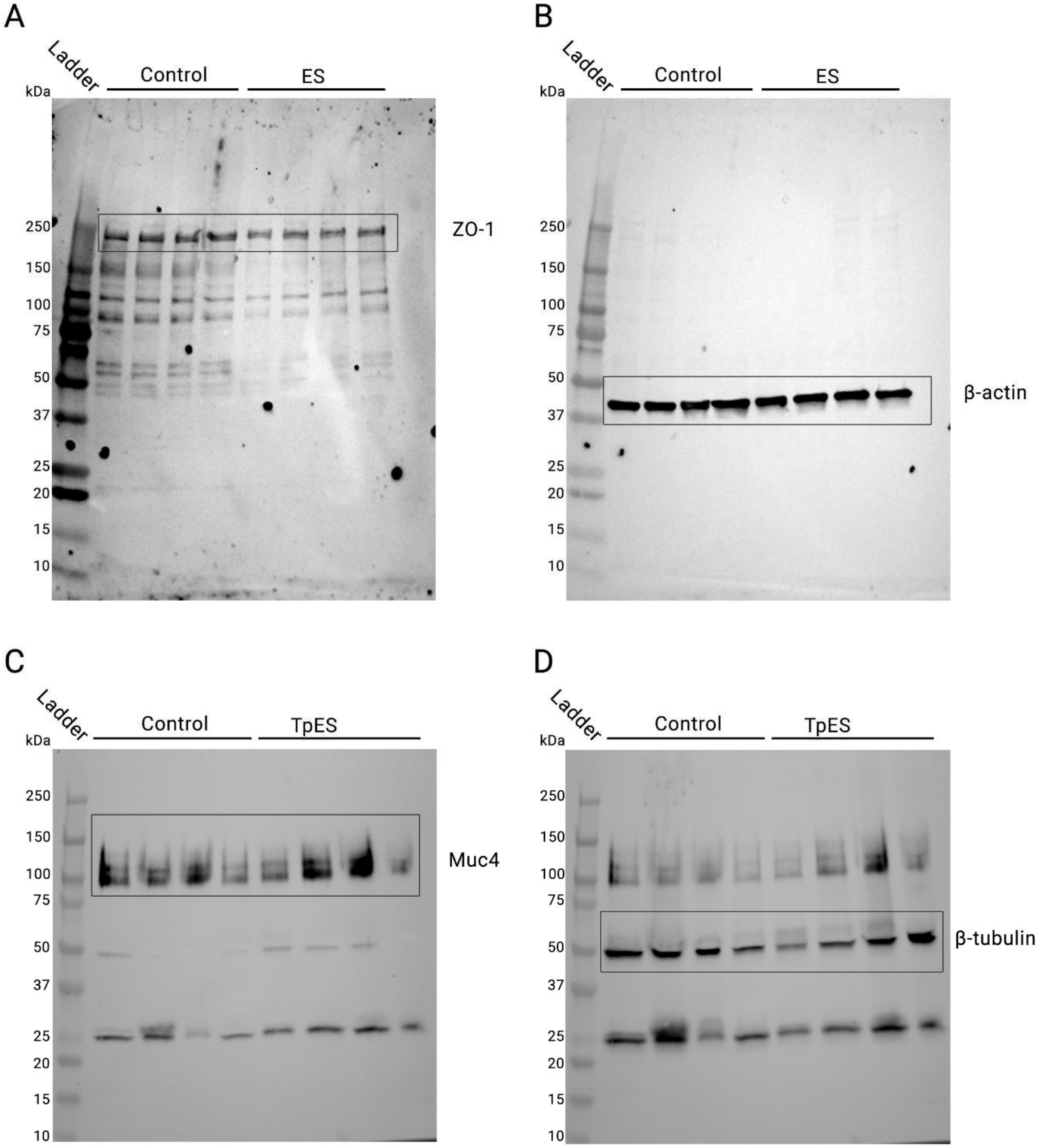

Supplementary Figure 3: Whole western blotting membrane images of (A) human ZO-1 (~260 kDa) and β-actin (~42 kDa) loading control (B). The cropped region used in Figure 3 is indicated by the black squares; (C) mouse Muc4 (~130 kDa) and (D) β-tubulin (~55 kDa) loading control.

The cropped region used in Figure 5 is indicated by the black squares. Images are acquired in automatic exposure determination before signal oversaturation.

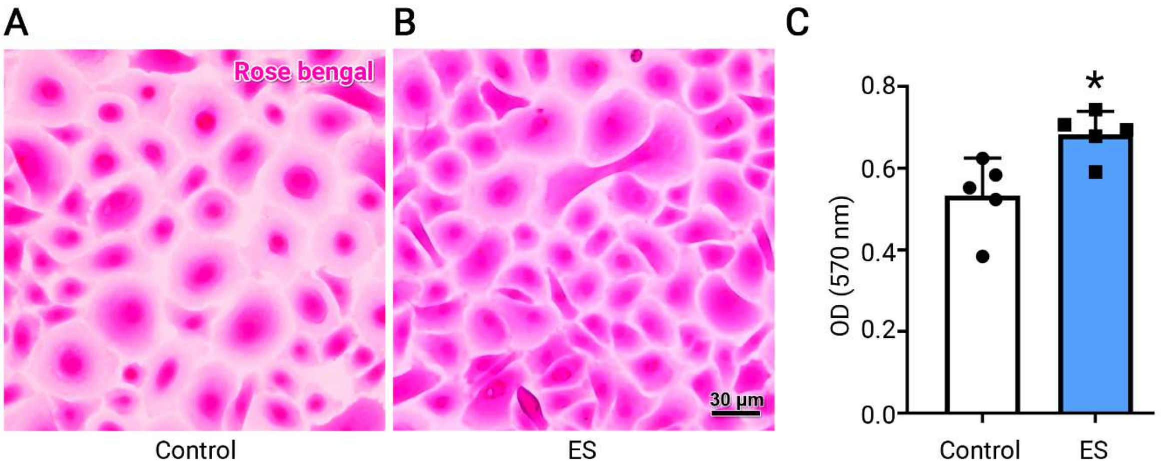

Supplementary Figure 4: Rose Bengal staining on primary corneal epithelial cells. Primary corneal epithelial cells were cultured on coverslips till confluency. ES was applied 30 min each day for 3 days and Rose Bengal 1 mg/ml was applied for 3 min, and images were taken under brightfield microscopy. (A) is a representative image of control and (B) is a representative image of cells treated with ES. After imaging, the cells were washed and Rose Bengal dye was extracted by methanol. The optical density values of methanol extracts were determined at 570 nm (C). n=5 (independent cultures) * $p < 0.05$.
